# Synthesis of 1,1,3,3,5,5-Hexamethyl-7,7-diorganocyclotetrasiloxanes and Its Copolymers

**DOI:** 10.3390/polym14010028

**Published:** 2021-12-22

**Authors:** Evgeniya V. Talalaeva, Aleksandra A. Kalinina, Evgeniy V. Chernov, Alina G. Khmelnitskaia, Marina A. Obrezkova, Georgii V. Cherkaev, Aziz M. Muzafarov

**Affiliations:** 1 Enikolopov Institute of Synthetic Polymeric Materials Russian Academy of Sciences (ISPM RAS), Profsoyuznaya 70, 117393 Moscow, Russia; talalaeva@ispm.ru (E.V.T.); kalinina@ispm.ru (A.A.K.); ak4erniY@gmail.com (E.V.C.); alina.khmelnitskaya@ispm.ru (A.G.K.); obrezkova@list.ru (M.A.O.); cherkaevgv@ispm.ru (G.V.C.); 2 A.N. Nesmeyanov Institute of Organoelement Compounds, Russian Academy of Sciences, Vavilov St., 28, 119991 Moscow, Russia

**Keywords:** mixed cyclosiloxanes, 1,1,3,3,5,5,7-heptamethyl-7-vinylcyclotetrasiloxane, 7-hydro-1,1,3,3,5,5,7-heptamethylcyclotetrasiloxane, 1,1,3,3,5,5,7-heptamethyl-7-phenylcyclotetrasiloxanes, 7,7-diethyl-1,1,3,3,5,5-hexamethylcyclotetrasiloxane, 1,1,3,3,5,5-hexamethyl-7,7-diphenylcyclotetrasiloxane, 1,5-disodiumoxyhexamethylsiloxane, poly(diethyl)(dimethyl)siloxane

## Abstract

This paper reports a method for the synthesis of 1,1,3,3,5,5-hexamethyl-7,7-diorganocyclotetrasiloxanes by the interaction of 1,5-disodiumoxyhexamethylsiloxane with dichlorodiorganosilanes such as methyl-, methylvinyl-, methylphenyl-, diphenyl- and diethyl dichlorosilanes. Depending on the reaction conditions, the preparative yield of the target cyclotetrasiloxanes is 55–75%. Along with mixed cyclotetrasiloxanes, the proposed method leads to the formation of polymers with regular alternation of diorganosylil and dimethylsylil units. For example, in the case of dichlorodiethylsilane, 70% content of linear poly(diethyl)dimethylsiloxanes with regular alternation of units can be achieved in the reaction product. Using 7,7-diethyl-1,1,3,3,5,5-hexamethylcyclotetrasiloxane as an example, the prospects of the mixed cycle in copolymer preparation in comparison with the copolymerization of octamethyl- and octaethylcyclotetrasiloxanes are shown.

## 1. Introduction

Cyclosiloxanes can be used as an initial reagent for the preparation of siloxane homo- and copolymer rubbers and liquids [1,2,3,4], as well as functional precursors for molecular design [5,6,7,8,9], cross-linking reagents [10], flame retardants [11], components of compositions for dry cleaning and detergents [12,13,14], solvents for coloring fabrics [15,16,17,18,19] and in cosmetics for various purposes, including skin and hair care products, deodorants/antiperspirants, makeup products, etc. [20,21,22,23].

Cyclosiloxanes of mixed composition are of particular interest in modifying polydimethylsiloxane [24,25,26,27] to provide it with the required properties and to obtain a linear functional matrix containing reactive groups in the chain for further transformations and obtaining new polymers with a determined structure and a required set of characteristics [28,29]. Substituents of the silicon atom have a strong effect on the polymerization rate of cyclosiloxanes; as a result, it is difficult to obtain copolymers by polymerization of a mixture of cyclosiloxanes with different groups at the silicon level [30,31,32]. This problem can be solved by using mixed dimethylcyclotetrasiloxanes. In this regard, the development of simple and effective methods for their preparation is an urgent task. Until very recently, no effective methods for the synthesis of mixed cyclotetrasiloxanes could be observed in the literature. For instance, mixed dimethylcyclotetrasiloxanes containing one silicon atom with different substituents can be synthesized via the cohydrolysis of dichlorodimethylsilane and the corresponding dichlorodiorganosilane, but the yield of the target cyclosiloxane does not exceed 30% [33,34,35]. Another approach is the heterofunctional condensation of hexamethyltrisiloxanes with terminal chloro- [36], hydro- [37,38] and hydroxy-groups [39,40] and the corresponding diorganosilanediols, diorganodialkoxy- or chlorosilanes. Mixed dimethylcyclotetrasiloxanes can form selectively using either diorganosilanediols or trisiloxanes with terminal functional groups. While the stability of diorganosilanediols limits the former approach [41,42,43,44], the multistep preparation and complexity of the method limit the latter [19,45,46,47,48].

Selective synthesis of 1,5-sodiumoxyhexamethyltrisiloxanes from dimethylsiloxanes of cyclic and linear structure [49] opens up opportunities for the directed production of mixed dimethylcyclotetrasiloxanes, including the transition to “green” chemistry methods. On one hand, the salt yield does not depend on the structure of the initial reagent; thus, low-molecular-weight debris from polydimethylsiloxane rubber production can become the raw material for its production. On the other hand, using an acceptor of hydrogen chloride, which is a standard component of hydrolytic and condensation processes with chlorsilanes and the presence of which has a significant effect on the cyclotetrasiloxane formation, is unnecessary [39,47].

Thus, this work aims to obtain mixed dimethylcyclotetrasiloxanes by reacting 1,5-sodiumoxyhexamethyltrisiloxanes with a number of dichlorodiorganosilanes. We further demonstrate the preparation of copolymers using dimethylcyclotetrasiloxanes.

## 2. Materials and Methods

### 2.1. Materials

The following reagents and organic solvents were used in the work: hexane, tetrahydrofuran (THF), methyl-*tret*-butyl ether (MTBE), anhydrous sodium hydroxide and potassium hydroxide from OOO “Component-Reaktiv”, Russia; methanol and pyridine from OOO “SpektrChem”, Russia; α,ω-dihydroxypolydimethylsiloxane brand SKTN A (PDMS) from OOO “Penta-91”, Russia; dichloromethylsilane 97%, dichloromethylsilane 97%, dichlorodiethylsilane 97%, dichloromethylphenylsilane 98%, chlorotrimethylsilane 97%, dichlorodiphenylsilane 98%, octamethylcyclotetrasiloxane, octaethylcyclotetrasiloxane from Reatorg, Russia.

All reagents were subjected to preliminary preparation in accordance with generally accepted methods [50]. Chlorosilanes were distilled immediately before use. Pyridine was dried over barium oxide. The toluene, THF and MTBE were distilled on a rotary evaporator and dried over calcium hydride.

1,5-disodiumoxyhexamethyltrisiloxane was obtained immediately before use by the interaction of sodium hydroxy and PDMS according to the procedure described in [51].

Synthesis of 1,1,3,3,5,5,7-heptamethyl-7-vinylcyclotetrasiloxane in MTBE medium (№ 1, Table 1). First, 20 g (0.07 mol) 1,5-disodiumoxyhexamethyltrisiloxane, 480 mL of anhydrous THF and 1 mL of anhydrous pyridine were added into a 2 L round-bottom flask equipped with a thermometer, reflux condenser and mechanical stirrer in an argon flow. Then, the reaction mass was heated to 66 °C with vigorous stirring and allowed to cool to room temperature. In this case, the salt completely dissolved and a clear solution was formed. After this, a solution of 11.9 g (0.08 mol) of dichloromethylvinylsilane in 268 mL of anhydrous THF was rapidly added to the reaction mixture and cooled to −60 °C with vigorous stirring. The reaction mass was stirred until room temperature (for 1 h). The pH of the reaction mass was 5–7. After this, the excess THF was distilled off on a rotary evaporator and MTBE was added. Then, the reaction mixture was washed with water to remove the precipitate, and the excess MTBE was distilled off on a rotary evaporator. The obtained siloxane product was analyzed by gas–liquid (GLC) and gel permeation chromatography (GPC). The results are shown in Table 1 (№ 1). Then, the product was distilled. As a result, 11.8 g containing of 98% of 1,1,3,3,5,5,7-hexamethyl-7-vinylcyclotetrasiloxane was isolated by 85 °C/20 mm Hg. The 1,1,3,3,5,5,7-hexamethyl-7-vinylcyclotetrasiloxane yield was 55%.

In addition, 7-hydro-1,1,3,3,5,5,7-heptamethylcyclotetrasiloxane, 7,7-diethyl-1,1,3,3,5,5-hexamethylcyclotetrasiloxane, 1,1,3,3,5,5,7-heptamethyl-7-phenylcyclotetrasiloxane and 1,1,3,3,5,5-hexamethyl-7,7-diphenylcyclotetrasiloxane were obtained analogously to this procedure in THF medium. The experimental results are presented in Table 1 (№ 3, 4, 5, 6, respectively).

Synthesis of 1,1,3,3,5,5,7-heptamethyl-7-vinylcyclotetrasiloxane in MTBE medium (№ 2, Table 1). First, 20 g (0.07 mol) 1,5-disodiumoxyhexamethyltrisiloxane, 341 mL of anhydrous THF and 1 mL of anhydrous pyridine were added into a 1 L round-bottom flask equipped with a thermometer, reflux condenser and mechanical stirrer in an argon flow. Then, the reaction mass was heated to 66 °C with vigorous stirring and allowed to cool to room temperature. A solution of 11.9 g (0.08 mol) of dichloromethylvinylsilane in 341 mL of anhydrous THF was prepared in a separate flask. Then, into another 2 L flask equipped with 2 reflux condensers, a thermometer and a mechanical stirrer, with vigorous stirring and cooling to −60 °C, a salt solution in THF and a solution of chlorosilane in THF were added simultaneously and at the same rate. The reaction mass was stirred until room temperature (for 1 h). The pH of the reaction mass was 5–7. After this, the excess THF was distilled off on a rotary evaporator and MTBE was added. Then, the reaction mixture was washed with water to remove the precipitate, and the excess MTBE was distilled off on a rotary evaporator. The obtained siloxane product was analyzed by GLC and GPC. The results are shown in Table 1 (№ 1). Then, the product was distilled. As a result, 9.9 g containing of 96% of 1,1,3,3,5,5,7-hexamethyl-7-vinylcyclotetrasiloxane was isolated. The 1,1,3,3,5,5,7-hexamethyl-7-vinylcyclotetrasiloxane yield was 45%.

Synthesis of 1,1,3,3,5,5,7-heptamethyl-7-vinylcyclotetrasiloxane in MTBE medium (№ 7, Table 1). First, 20 g (0.07 mol) 1,5-disodiumoxyhexamethyltrisiloxane, 577 mL of anhydrous MTBE and 1 mL of anhydrous pyridine were added into a 2 L round-bottom flask equipped with a thermometer, dropping funnel and mechanical stirrer in an argon flow. A solution of 11.9 g (0.08 mol) of dichloromethylvinylsilane in 322 mL of anhydrous MTBE was rapidly added to the reaction mixture and cooled to −60 °C with vigorous stirring. The reaction mass was stirred until room temperature (for 1 h). The pH of the reaction mass was 5-7. Then, the reaction mixture was washed with water to remove the precipitate, and the excess MTBE was distilled off on a rotary evaporator. The obtained siloxane product was analyzed by GLC and GPC. The results are shown in Table 1 (№ 1). Then, the product was distilled. As a result, 16.3 g containing of 97% of 1,1,3,3,5,5,7-hexamethyl-7-vinylcyclotetrasiloxane was isolated by 85 °C/20 mm Hg. The 1,1,3,3,5,5,7-hexamethyl-7-vinylcyclotetrasiloxane yield was 75%.

In addition, 7-hydro-1,1,3,3,5,5,7-heptamethylcyclotetrasiloxane, 7,7-diethyl-1,1,3,3,5,5-hexamethylcyclotetrasiloxane, 1,1,3,3,5,5,7-heptamethyl-7-phenylcyclotetrasiloxane and 1,1,3,3,5,5-hexamethyl-7,7-diphenylcyclotetrasiloxane were obtained analogously to this procedure in MTBE medium. The experimental results are presented in Table 2 (№ 9, 10, 11, 12, respectively).

Synthesis of 1,1,3,3,5,5,7-heptamethyl-7-vinylcyclotetrasiloxane in MTBE medium (№ 8, Table 1). First, 11.9 g (0.08 mol) of dichloromethylvinylsilane, in 899 mL of anhydrous MTBE, and 1 mL of anhydrous pyridine were added into a 2 L round-bottom flask equipped with a thermometer, dropping funnel and mechanical stirrer in an argon flow. Then, 20 g (0.07 mol) dry 1,5-disodiumoxyhexamethyltrisiloxane was rapidly added to the reaction mixture and cooled to −60 °C with vigorous stirring. The reaction mass was stirred until room temperature (for 1 h). The pH of the reaction mass was 5–7. Then, the reaction mixture was washed with water to remove the precipitate, and the excess MTBE was distilled off on a rotary evaporator. The obtained siloxane product was analyzed by GLC and GPC. The results are shown in Table 1 (№ 1). Then, the product was distilled. As a result, 15.2 g containing of 97% of 1,1,3,3,5,5,7-hexamethyl-7-vinylcyclotetrasiloxane was isolated. The 1,1,3,3,5,5,7-hexamethyl-7-vinylcyclotetrasiloxane yield was 70%.

All obtained cycles were characterized by ^1^H and ^29^Si nuclear magnetic resonance (NMR)-1,1,3,3,5,5,7-hexamethyl-7-vinylcyclotetrasiloxane 
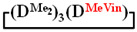
. ^1^H NMR, δ, ppm: 5.67–6.19 m (3H, ((CH_2_=CH)Si), 0.15 m (12H, Si(CH_3_)_2_). ^29^Si NMR, δ ppm: −18.48 (2Si, Si(CH_3_)_2_O_2/2_), −18.89 (1Si, Si(CH_3_)_2_O_2/2_), −33.47 (1Si, Si(CH_3_)(CH_2_=CH)O_2/2_).

7-hydro-1,1,3,3,5,5,7-heptamethylcyclotetrasiloxane 
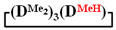
. ^1^H NMR, δ, ppm: 4.70 s (1H, SiH), 0.15 m (21H, Si(CH_3_)_2_). ^29^Si NMR, δ, ppm: −17.65 (2Si, Si(CH_3_)_2_O_2/2_), −18.79 (1Si, Si(CH_3_)_2_O_2/2_), −34.79 (1Si, Si(CH_3_)(H)O_2/2_).

7,7-diethyl-1,1,3,3,5,5-hexamethylcyclotetrasiloxane 
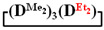
 ^1^H NMR, δ, ppm: 0.91–0.96 t (6H, Si(CH_2_CH_3_)_2_), 0.49–0.52 q (4H, Si(CH_2_CH_3_)_2_), 0.08–0.10 d (18H (Si(CH_3_)_2_). ^29^Si NMR, δ ppm: −19.24 (1Si, Si(CH_2_CH_3_)_2_O_2/2_), −19.45 (1Si, Si(CH_3_)_2_O_2/2_), −19.58 (2Si, Si(CH_3_)_2_O_2/2_).

1,1,3,3,5,5,7-heptamethyl-7-phenylcyclotetrasiloxane 
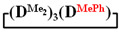
. ^1^H NMR, δ, ppm: m 7.25-7.57 (5H, Si(C_6_H_5_)), 0.24 s (3H, Si(CH_3_)), 0.03–0.07 m (18H, Si(CH_3_)_2_). ^29^Si NMR, δ ppm: −18.07 (2Si, Si(CH_3_)_2_O_2/2_), −18.61 (1Si, Si(CH_3_)_2_O_2/2_), −32.59 (1Si, Si(CH_3_)(C_6_H_5_)O_2/2_).

1,1,3,3,5,5-hexamethyl-7,7-diphenylcyclotetrasiloxane 
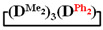
. ^1^H NMR, δ, ppm: 7.25–7.58 m (10H, Si(C_6_H_5_)_2_), 0.01–0.06 m (18H, (Si(CH_3_)_2_). ^29^Si NMR, δ ppm: −17.49 (2Si, Si(CH_3_)_2_O_2/2_), −18.51 (1Si, Si(CH_3_)_2_O_2/2_), −46.19 (1Si, Si(C_6_H_5_)_2_O_2/2_).

Polymerization of 
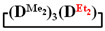
. First, 5 g (0.015 mol) of 7,7-diethyl-1,1,3,3,5,5-hexamethylcyclotetrasiloxane and 0.16 g (2.85 mmol) of KOH were stirred at 140 °C for 1 h. At the end of the polymerization, a colorless, highly viscous product was obtained. After this, at 5 °C, 13.5 mL anhydrous toluene, 1.5 g (0.014 mol) of chlorotrimethylsilane and 1.1 g (0.014 mol) pyridine were added to the reaction mixture. The resulting product was washed to neutral pH of the aqueous layer, and the solution was dried over anhydrous sodium sulfate. Then, the excess solvent was removed on a rotary evaporator and the polymer was dried at 1 mmHg. The resulting product was characterized by a bimodal molecular weight distribution. The high-molecular-weight part was separated using preparative gel permeation chromatography. The product was analyzed by GPC and NMR methods. The copolymerizations of octamethylcyclotetrasiloxane 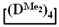 with octaethylcyclotetrasiloxane 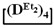 or 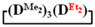 were performed in a similar manner to this procedure. The copolymerization conditions and results are shown in Table 3.

### 2.2. Methods

GLC analysis was performed on a Chromatek Analytic 5000 chromatograph (Russia), a katharometer detector, a helium carrier gas, 2 m × 3 mm columns and a stationary phase SE-30 (5%) printed on Chromaton-H-AW. Registration and calculation of data were carried out using the program “Chromatek Analyst” (Russia).

GPC analysis was performed on a chromatographic system consisting of a STAYER series 2 high-pressure pump (Aquilon, Russia), a RIDK 102 refractometric detector (Czech Republic) (using eluent—toluene) and a JETSTREAM 2 PLUS column thermostat (KNAUER, Berlin, Germany). Eluents—toluene + 2% THF, flow rate—1.0 mL/min. Columns 300 mm long and 7.8 mm in diameter (300 × 7.8 mm) were filled with the Phenogel sorbent (Phenomenex, Torrance, CA, USA), the particle size was 5 mm, and the pore size was 10^3^A and 10^4^A (the passport separation range was up to 75,000 Da and up to 500,000 Da, respectively). The registration and calculation of data were performed using the UniChrom 4.7 program (Belarus).

^1^H and ^29^Si NMR spectra of products were recorded using a Bruker Avance II 300 spectrometer. CDCl_3_ was used as the internal standard with a chemical shift of δ = 7.25 ppm.

Infrared (IR) spectra were recorded on an IR Fourier spectrometer—Nicolet iS50 (Thermo Scientific, Waltham, MA, USA)—with a built-in ATR (crystal-diamond) attachment. Measurement conditions: resolution—4 cm^−1^, number of scans—32.

Differential scanning calorimetry (DSC) of samples was performed on the differential scanning calorimeter DSC-3 (Mettler-Toledo, Switzerland) at a heating rate of 10°/min in an argon atmosphere (60 mL/min).

## 3. Results and Discussion

### 3.1. Synthesis of 1,1,3,3,5,5-Hexamethyl-7,7-diorganocyclotetrasiloxanes

**Figure 1:**
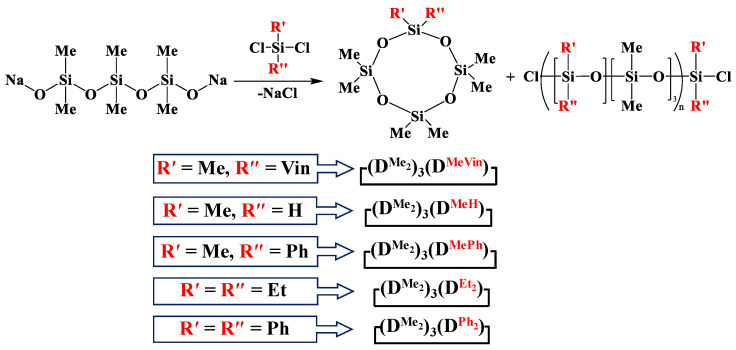
Scheme of interaction of 1,5-disodiumoxyhexamethyltrisiloxane and diorganodichlorosilanes.

The general scheme of interaction of 1,5-sodiumoxyhexamethyltrisiloxane with dichlorodiorganosilanes is shown in Figure 1.

Methylvinyl- and methyldichlorosilanes were used as dichlorodiorganosilane for the synthesis of functional mixed dimethylcyclotetrasiloxanes. To study the effect of the substituent type, the interaction of 1,5-disodiumoxyhexamethyltrisiloxane and methylphenyl-, diphenyl- and diethyldichlorosilanes was also investigated.

Firstly, 1,5-disodiumoxyhexamethyltrisiloxane is a white hygroscopic powder, practically insoluble in most organic solvents. Its dissolution in tetrahydrofuran or pyridine is achieved only at temperatures up to 50–60 °C, but, even in this case, the solubility of the salt does not exceed 5 wt.%. Therefore, it was of interest to compare the process under homogeneous and heterogeneous conditions at the same concentration of reagent (5 wt.%) in the reaction mixture. THF was used for homogeneous conditions; MTBE was used for heterogeneous conditions. Regardless of other conditions, the reaction was carried out at −60 °C to prevent the processes of cleavage of the siloxane bond under the action of silanolate end groups. The reaction mixture was intensively stirred for 1 h after adding the reagents. If the pH was neutral or slightly acidic, the reaction mixture was stirred until room temperature. The siloxane product was isolated and analyzed by GPC, and the 1,1,3,3,5,5-hexamethyl-7,7-diorganocyclotetrasiloxane was isolated by distillation at reduced pressure. The purity and structure of the mixed cyclotetrasiloxanes were confirmed by a combination of GLC and ^1^H and ^29^Si NMR spectroscopy methods. The reaction conditions and the composition of the products are shown in Table 1 and Table 2.

**Figure 2:**
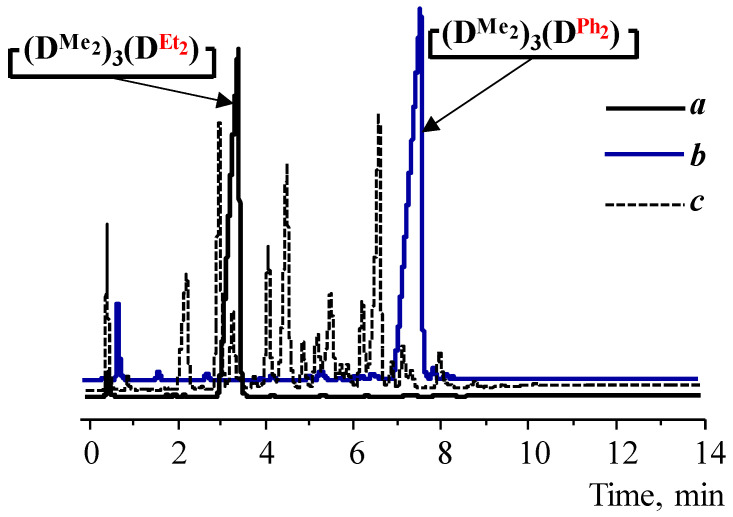
GLC curves of volatile products 4 (a) (Table 1), 12 (b) (Table 2), obtained at the reaction temperature of −60 °C, and the product of the interaction of the salt with dichlorodiethylsilane at room temperature.

GLC data indicate the absence of side processes with the silanolate ends’ participation. In all cases, depending on the type of diorganodichlorosilane and solvent, volatile products consisted of the target cyclotetrasiloxane by 85–98% (Figure 2a,b, Table 1 and Table 2). In comparison, Figure 2c shows the GLC curve of the volatile products of the 1,5-disodiumoxyhexamethyltrisiloxane and dichlorodiethylsilane interaction under homogeneous conditions, where the processes of siloxane bond cleavage and rearrangement of the resulting products were found.

**Figure 3:**
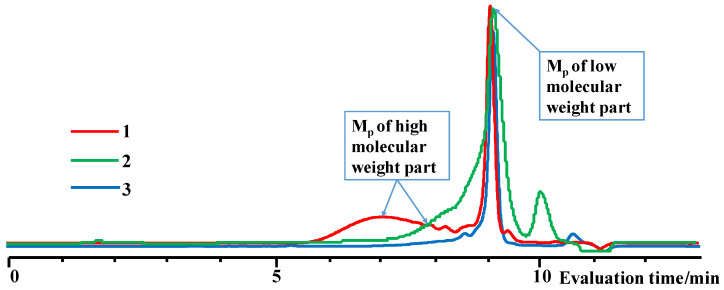
GPC curves of products 1, 2, 7 (Table 1 and Table 2).

The effect of adding reagents on the reaction mixture was studied in the case of dichloromethylvinylsilane and 1,5-disodiumoxyhexamethyltrisiloxane. It was found that the order of reagent injection under homogeneous conditions and heterogeneous conditions did not significantly affect the yield of the target 
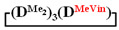
. Thus, under homogeneous conditions, the target cycle was formed in 45–55% yield, both when adding chlorosilane in THF to a solution of salt in THF (№ 1, Table 1) and with the simultaneous injection solutions of salt and chlorosilane in THF with the same molarity (№ 2, Table 1). In this case, the sequence of reagent addition affected only the molecular weight distribution of linear oligomers (Figure 3). Under heterogeneous conditions, salt was added to a chlorosilane in MTBE (№ 4, Table 1) or chlorosilane to a suspension of salt in MTBE, and the yield of the product was 70–75% (№ 8, Table 1).

Further interactions were carried out by adding a solution of dichlorodiorganosilane to a solution or suspension of the salt in THF or MTBE, respectively.

Analysis of the data in Table 1 and Table 2 allowed us to divide all cases into two groups. Vinylmethyl- and methyldichlorosilanes showed the highest preparative yield of 
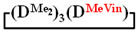
 and 
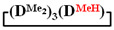
 in MTBE, which is 55 and 75%, respectively (№ 3 and 6, Table 1). The opposite situation was observed using more sterically hindered chlorosilyl end groups such as diethyl-, methylphenyl- and diphenyldichlorosilanes: the highest yields of 
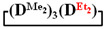
, 
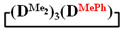
 and 
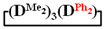
 were achieved under homogeneous conditions, equal to up to 65, 67 and 70%, respectively (№ 8, 10, 12, Table 2). Such differences in the yields of products indicate significant opportunities for further optimization of the yield of each specific mixed cycle.

All dimethylcyclotetrasiloxanes were isolated with a purity of at least 95% according to GLC data; the structure of the obtained products was confirmed by ^1^H, ^29^Si NMR and IR spectroscopy. The relevant data are given in the Supplementary Materials (Figures S1–S15). The IR spectroscopy data of the isolated cycles indicate the absence of an absorption band in the region of 3400–3600 cm^−1^, which is characteristic of silanol groups and confirms the cyclic structure of the isolated compounds (Figures S1–S5). The ^1^H and ^29^Si NMR spectroscopy data of the isolated fractions indicate that the integral intensities of the protons signals of corresponding substituents at silicon atoms and silicon atoms themselves conform to the calculated values (Figures S11–S15).

**Figure 4:**
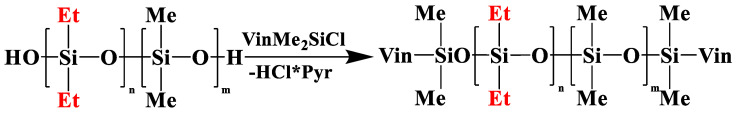
Scheme of blocking linear poly(diethyl)(dimethyl)siloxane (№ 10, Table 2).

**Figure 5:**
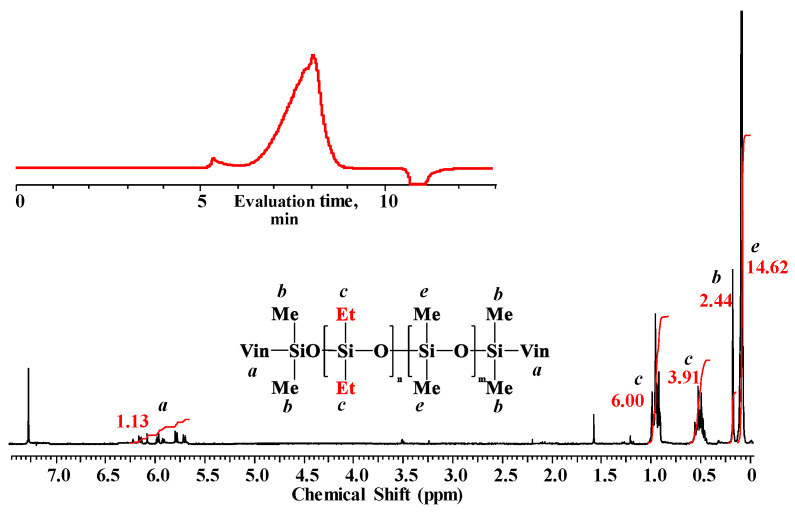
^1^H NMR spectrum and GPC curve of blocked linear poly(diethyl)(dimethyl)siloxane (№ 10, Table 2).

The data in Table 1 and Table 2 show that the main reaction product may be a linear oligomer with a regular arrangement of modifying units under certain conditions. In particular, in sample 10 (Table 2), the product contained 
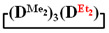
 along with the linear poly(diethyl)(dimethyl)siloxane with Mp = 1900 and content of 70%. The product was blocked with chlorodimethylvinylsilane to confirm the linear structure (Figure 4) and its composition and molecular weight characteristics were determined by ^1^H NMR spectroscopy and GPC methods (Figure 5).

The correlation of the integral intensities of proton signals of ethyl, vinyl and methyl groups in the backbone and terminal silicon atoms allowed us to determine by the ^1^H NMR spectrum that the unit composition of the obtained product corresponded to the following formula: VinMe_2_SiO-{[Et_2_SiO]_1_[Me_2_SiO]_2,4_}_5,3_-SiMe_2_Vin with M_n_ equal to ~1640. The number-average molecular weights of the polymer calculated from the NMR and determined by the GPC method (M_n_ = 1800, M_w_ = 2300, M_w_/M_n_ = 1.3) were consistent and confirmed the linear structure of poly(diethyl)(dimethyl)siloxane.

Thus, the interactions of 1,5-disodiumoxyhexamethyltrisiloxane with diorganodichlorosilanes were investigated to obtain 1,1,3,3,5,5-hexamethyl-7,7-diorganocyclotetrasiloxanes. For the first time, it was shown that mixed dimethylcyclotetrasiloxanes can be obtained with a yield of 55 to 75% by this method. The ratio of linear and cyclic products of a mixed structure can be controlled within wide limits by selecting the reaction conditions. Using dichlorodiethylsilane as an example, it was shown that this method can be a promising means of obtaining linear oligomers with alternating diethyl- and dimethylsiloxane units.

### 3.2. Preparation of Poly(diethyl)(dimethyl)siloxane

**Figure 6:**
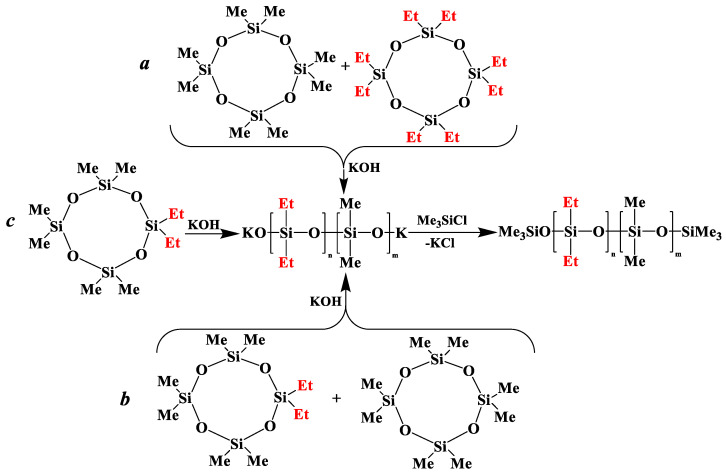
Polymerization schematics of 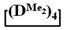 and 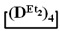 (**a**), of 
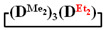
 and 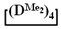 (**b**) and of 
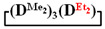
 (**c**).

A simple and cheap method for the preparation of 1,1,3,3,5,5-hexamethyl-7,7-diorganocyclotetrasiloxanes opens up new prospects for the preparation of polydimethyldiorganosiloxanes with a controlled content of diorganosilyl groups via polymerization methods. It is known that, in order to obtain polydiethylsiloxanes, hexaethylcyclotrisiloxane is polymerized [51,52] since octaethylcyclotetrasiloxane 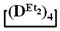 is practically not polymerized. To obtain poly(diethyl)(dimethyl)siloxane copolymers, catalytic rearrangement of the cohydrolysis products of dimethyl- and diethyldichlorosilanes is carried out [53]. In our study, we paid attention to the prospects of using mixed 
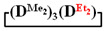
, in contrast to 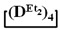, for the preparation of (diethyl)(dimethyl)siloxane copolymers. Anionic polymerization of 
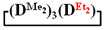
, its copolymerization with 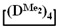 and copolymerization of 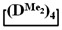 and 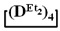 in the presence of potassium hydroxide were carried out to illustrate this statement (Figure 6a–c, respectively). The duration of anionic polymerization was 1 h at 140 °C. Trimethylchlorosilane was used as a termination agent.

**Figure 7:**
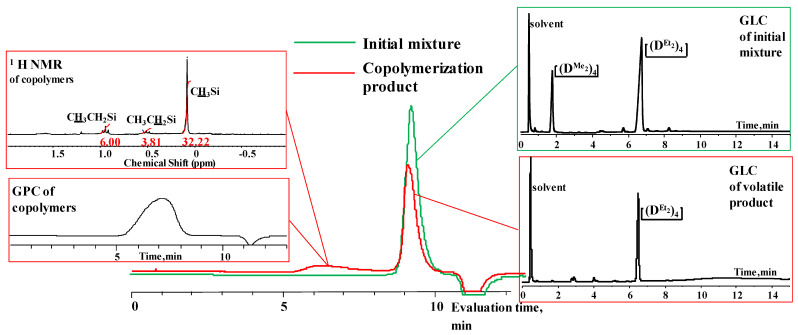
Characteristics of the initial mixture of 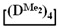/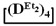 and the product of their copolymerization (№ 1, Table 3): GPC data for the initial mixture (green curve) and the product (red curve); GLC curves of the initial mixture of monomers (top right) and volatile fraction after copolymerization (bottom right); ^1^H NMR spectrum (top left) and GPC curve (bottom left) of obtained copolymer.

**Figure 8:**
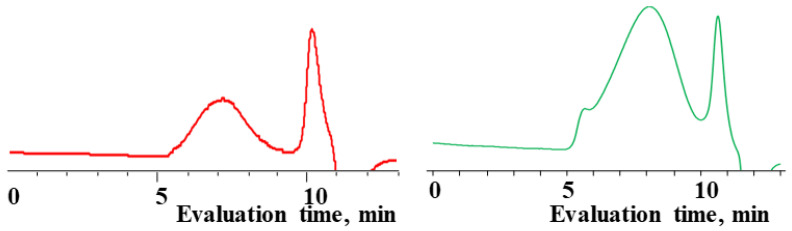
GPC curves of copolymerization 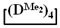 and 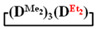 (on left) and polymerization 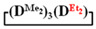 (on right) products.

The content of the high-molecular and low-molecular parts of the products was determined by the GPC method (Table 3, Figure 7 and Figure 8). The high-molecular-weight part was separated using preparative GPC, and its composition and molecular weight characteristics were analyzed by ^1^H NMR spectroscopy and GPC methods (Figures S16–S18). The characteristics of the obtained products are shown in Table 3.

As expected, the content of the high-molecular part was three times higher in the case of the copolymerization of 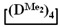 and 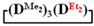 (№ 2, Table 3) than in the copolymerization of homocycles 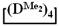 and 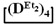 with various substituents (№ 1, Table 3), where low conversion of the 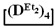 was observed. It follows from a comparison of the GLC data for the initial mixture of monomers and the low-molecular-weight fraction of the products (Figure 7). Analysis of the high-molecular-weight fractions of the products showed the correspondence of the structural unit of the copolymer obtained by the copolymerization of 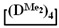 and mixed 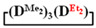 to the calculated value, in contrast to the copolymerization of 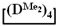 and 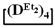, where the polymer composition was enriched with dimethylsilyl units.

The polymerization of mixed 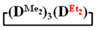 forms poly(diethyl)dimethylsiloxane with a Mn close to the calculated value, a broad molecular weight distribution and a structural unit composition corresponding to the calculated one (№3, Table 3, Figure 8 (on right)). According to DSC data (Figures S19 and S20), the obtained poly(diethyl)dimethylsiloxanes (№ 2 and 3 of Table 3) had a low glass transition temperature of −132 °C~–131 °C and the absence of crystallization.

Thus, firstly, the advantages of 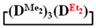 used for the preparation of poly(diethyl)(dimethyl)siloxanes with a controlled unit composition were demonstrated in comparison with the mixture of 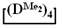 and 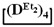.

## 4. Conclusions

Mixed tetrasiloxane cycles have high potential for practical application; however, the lack of selective methods for its preparation has been a limiting factor for the realization of this potential for a long time. This work shows that high selectivity of mixed cycle synthesis can be achieved based on 1,5-disodiumoxyhexamethyltrisiloxanes, a unique reagent that we described earlier [49]. The yield of these cyclosiloxanes in the best experiments reaches 70%. It is important that linear alternating oligomers are formed as by-products, which can be used independently. Moreover, the ratio between linear and cyclic products can be changed within wide limits.

The second part of the article demonstrates the advantages of mixed cyclosiloxane polymerization in comparison with a mixture of two cyclosiloxanes with a homogeneous structure. This result is a consequence of the low reactivity of 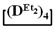 in comparison with the high reactivity mixed cycle in anionic polymerization.

We believe that the considered method opens up new prospects both for expanding the range of cyclic siloxane products with a specific composition and structure, which have many different applications, and for obtaining linear polymers with a controlled content of modifying units and new materials based on them. Mixed cycles have many other applications, including fluids with controlled properties. The realization of these and other potential applications requires further research and is an important subject of current research.

## Figures and Tables

**Table 1:** Reaction conditions in THF and product characteristics.

№	Target Cyclotetrasiloxane	Sequence of Reagent Addition	Characteristics of Products					Preparative Yield of Cycle, %
			The Yield of Target Cycle in Volatile Products by GLC, %	GPC Data				
				Low-Molecular-Weight Part		High-Molecular-Weight Part		
				%	M_p_	%	M_p_	
1	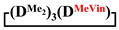	Cl→ONa ^1^	85	50	500	50	3700	55
2		Cl║ONa ^2^	75	55	500	45	1000	45
3	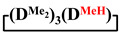	Cl→ONa	86	40	500	60	5000	45
4	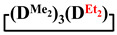	Cl→ONa	98	60	700	40	2100	65
5	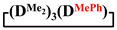	Cl→ONa	99	80	700	20	1800	67
6	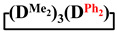	Cl→ONa	97	70	700	30	900	70

^1^ Rapid injection of a solution of diorganodichlorosilane to a solution or suspension of 1,5-disodiumoxyhexamethyltrisiloxane; ^2^ simultaneous mixing of solutions of diorganodichlorosilane and 1,5-disodiumoxyhexamethyltrisiloxane with the same molarity.

**Table 2:** Reaction conditions in MTBE and product characteristics.

№	Target Cyclotetrasiloxane	Sequence of Reagent Addition	Characteristics of Products					Preparative Yield of Cycle, %
			The Yield of Target Cycle in Volatile Products by GLC, %	GPC Data				
				Low-Molecular-Weight Part		High-Molecular-Weight Part		
				%	M_p_	%	M_p_	
7	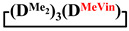	Cl→ONa	85	90	500	10	800	75
8		ONa→Cl ^3^	83	80	500	20	1000	70
9	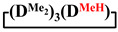	Cl→ONa	97	40	500	60	2400	55
10	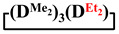	Cl→ONa	80	30	700	70	1900	30
11	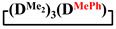	Cl→ONa	80	70	700	30	1200	40
12	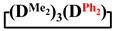	Cl→ONa	90	60	700	40	900	38

^3^ Adding dry 1,5-disodiumoxyhexamethyltrisiloxane to the diorganodichlorosilane solution.

**Table 3:** Polymerization conditions and characteristics of products.

№	Monomer Ratio, mol/mol	KOH ^1^, mol	M_n_ Theor.	Molecular Weight Characteristics of the Product (GPC)					Et_2_SiO/ Me_2_SiO, mol/mol		T_g_/T_c_, °C
				% Low-Molecular-Weight Part	% High-Molecular-Weight Part	M_w_	M_n_	M_w_/M_n_	Calc.	NMR	
1	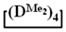 / 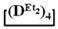 1/1	0.032	11,000	80	20	16,200	8300	1.9	1/1	1/5.4	unchanged
2	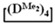 / 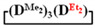 1/1	0.032	10,000	40	60	68,200	30,300	2.3	1/7	1/6.0	−131/−
3	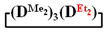	0.20	1700	17	83	195,000	1400	140	1/3	1/3.3	−132/−

^1^ The amount of alkali per 1 mol of monomer.

## Supplementary Materials

The following supporting information can be downloaded at: https://www.mdpi.com/article/10.3390/polym14010028/s1, Figures S1–S5: IR spectra of all obtained cycles; Figures S6–S10: GLC curves of all obtained cycles; Figures S11–S15: ^1^H (top) and ^29^Si (bottom) NMR spectra for mixed cyclotetrasiloxanes; Figures S16–S18: ^1^H NMR spectra for high-molecular-weight copolymers; Figures S19 and S20: DSC thermograms for high-molecular-weight copolymers.
