# Supplementary material for: Synthesis of 1,1,3,3,5,5-Hexamethyl-7,7-diorganocyclotetrasiloxanes and Its Copolymers
Source: Polymers (Basel). 2021 Dec 22;14(1):28. doi: 10.3390/polym14010028 (PMC8747541; doi:10.3390/polym14010028)
Supplement: Supplementary file 1 [file polymers-14-00028-s001.zip › polymers-1488327-SI.pdf]

polymers
MDPI

Supplementary materials:

Synthesis of 1,1,3,3,5,5-Hexamethyl-7,7-diorganocyclotetrasiloxanes and its Copolymers

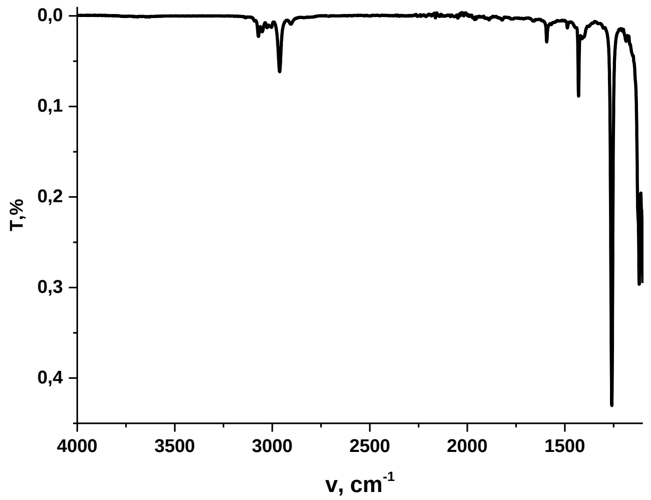

Figure S1. IR spectra of 1,1,3,3,5,5,7-heptamethyl-7-vinylcyclotetrasiloxane

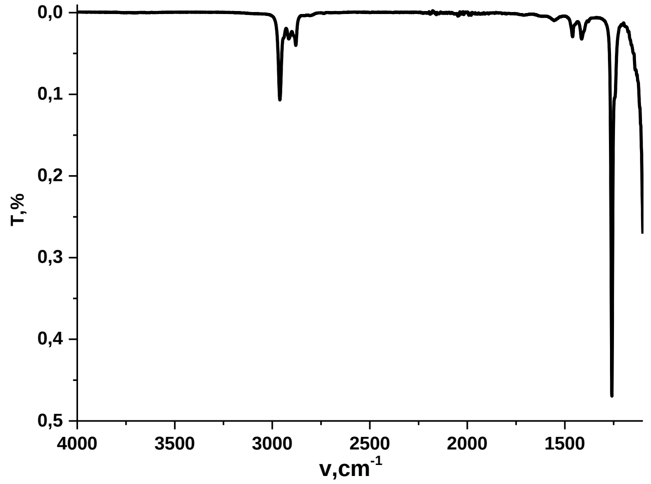

Figure S2. IR spectra of 7,7-diethyl-1,1,3,3,5,5-hexamethylcyclotetrasiloxane

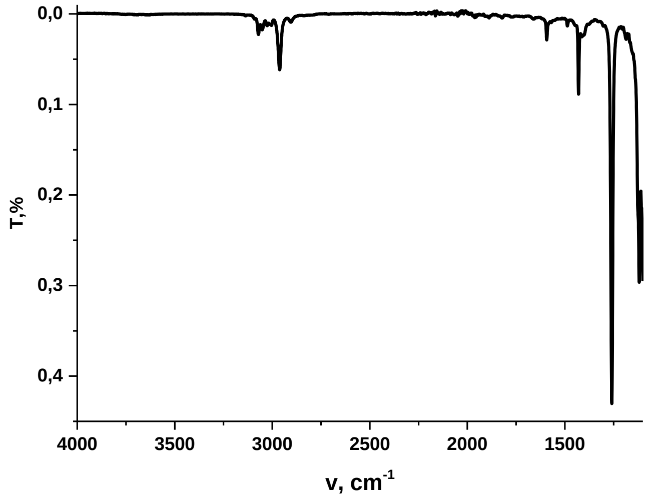

Figure S3. IR spectra of 1,1,3,3,5,5-hexamethyl-7,7-diphenylcyclotetrasiloxane

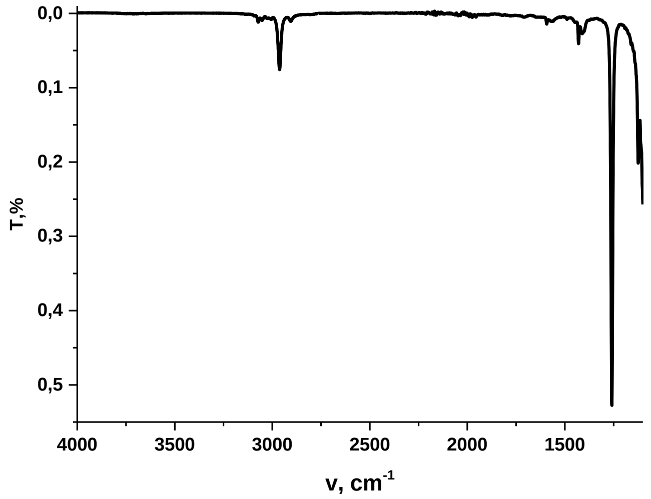

Figure S4. IR spectra of 1,1,3,3,5,5,7-heptamethyl-7-phenylcyclotetrasiloxane

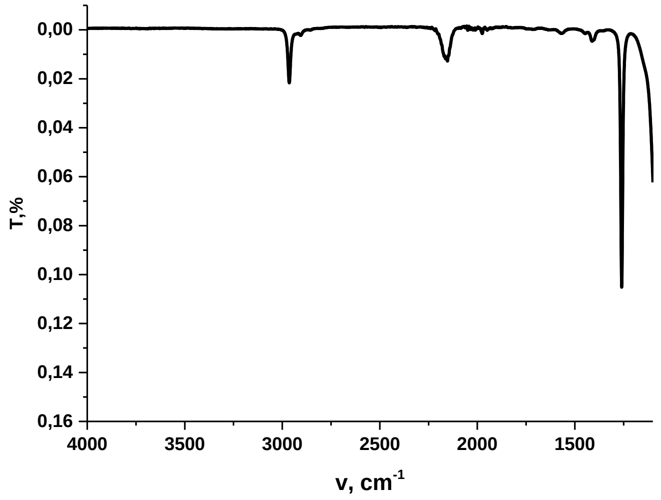

Figure S5. IR spectra of 7-hydro-1,1,3,3,5,5,7-heptamethylcyclotetrasiloxane

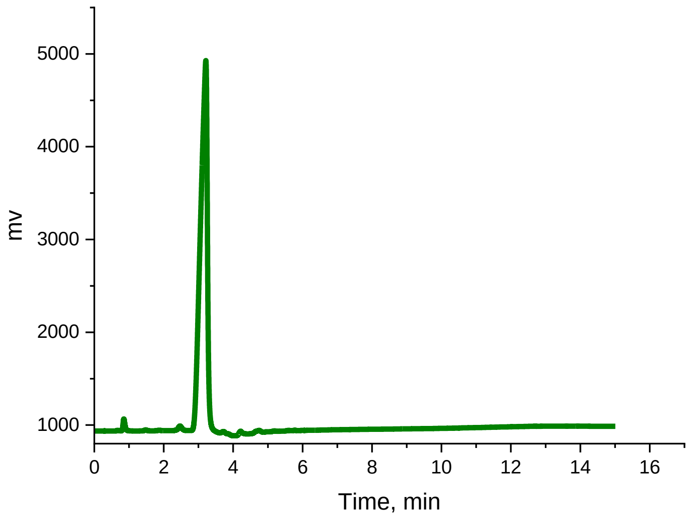

Figure S6. GLC curve of 1,1,3,3,5,5,7-heptamethyl-7-vinylcyclotetrasiloxane

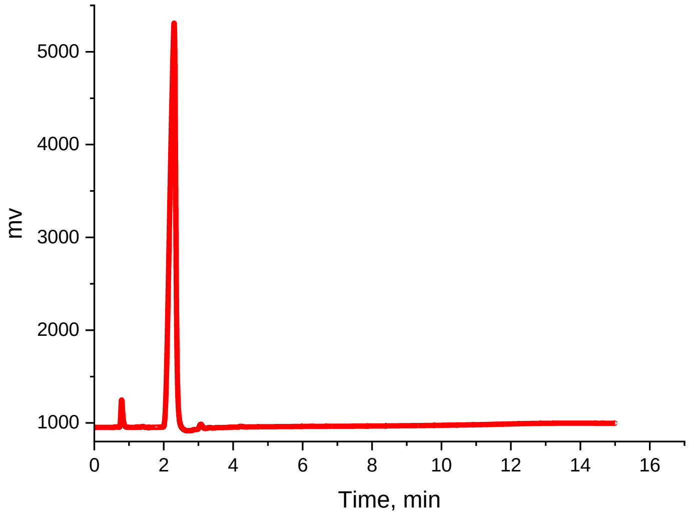

Figure S7. GLC curve of 7-hydro-1,1,3,3,5,5,7-heptamethylcyclotetrasiloxane

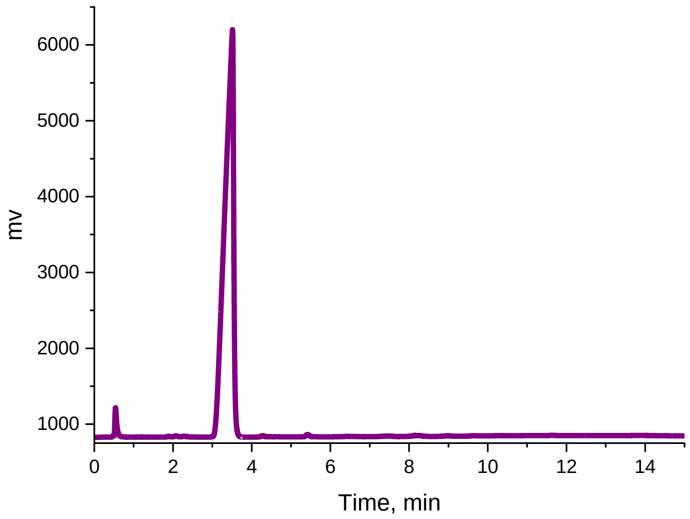

Figure S8. GLC curve of 7,7-diethyl-1,1,3,3,5,5-hexamethylcyclotetrasiloxane

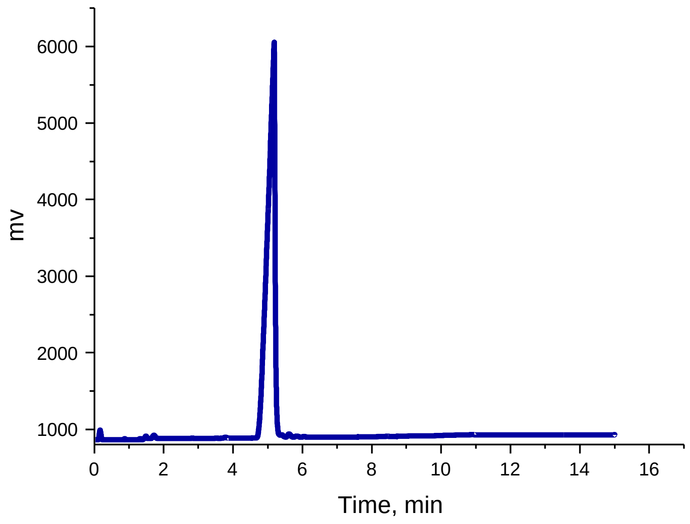

Figure S9. GLC curve of 1,1,3,3,5,5,7-heptamethyl-7-phenylcyclotetrasiloxane

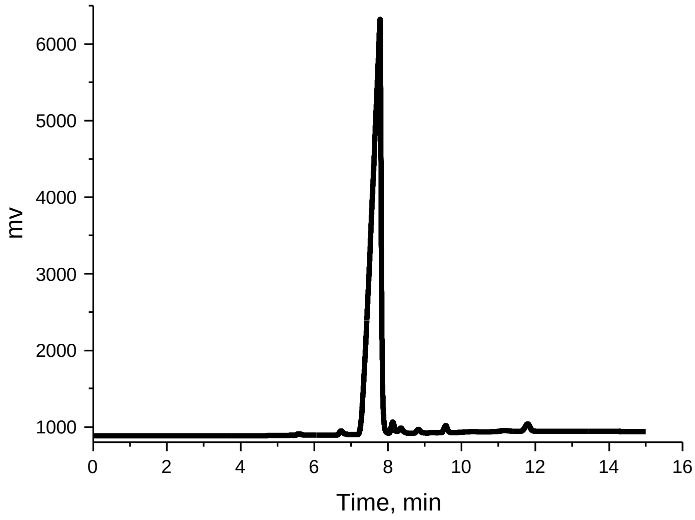

Figure S10. GLC curve of 1,1,3,3,5,5-hexamethyl-7,7-diphenylcyclotetrasiloxane

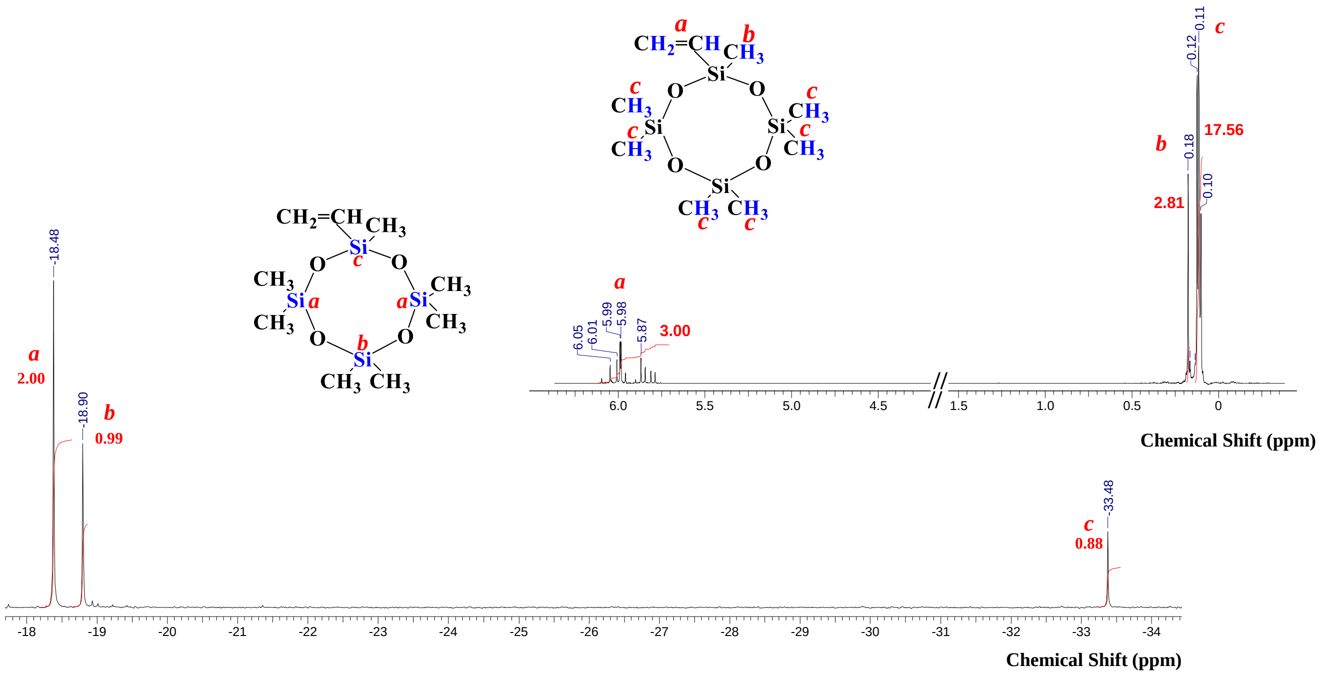

Figure S11. ^{1}H (top) and ^{29}Si (bottom) NMR spectra for 1,1,3,3,5,5,7-heptamethyl-7-vinylcyclotetrasiloxane

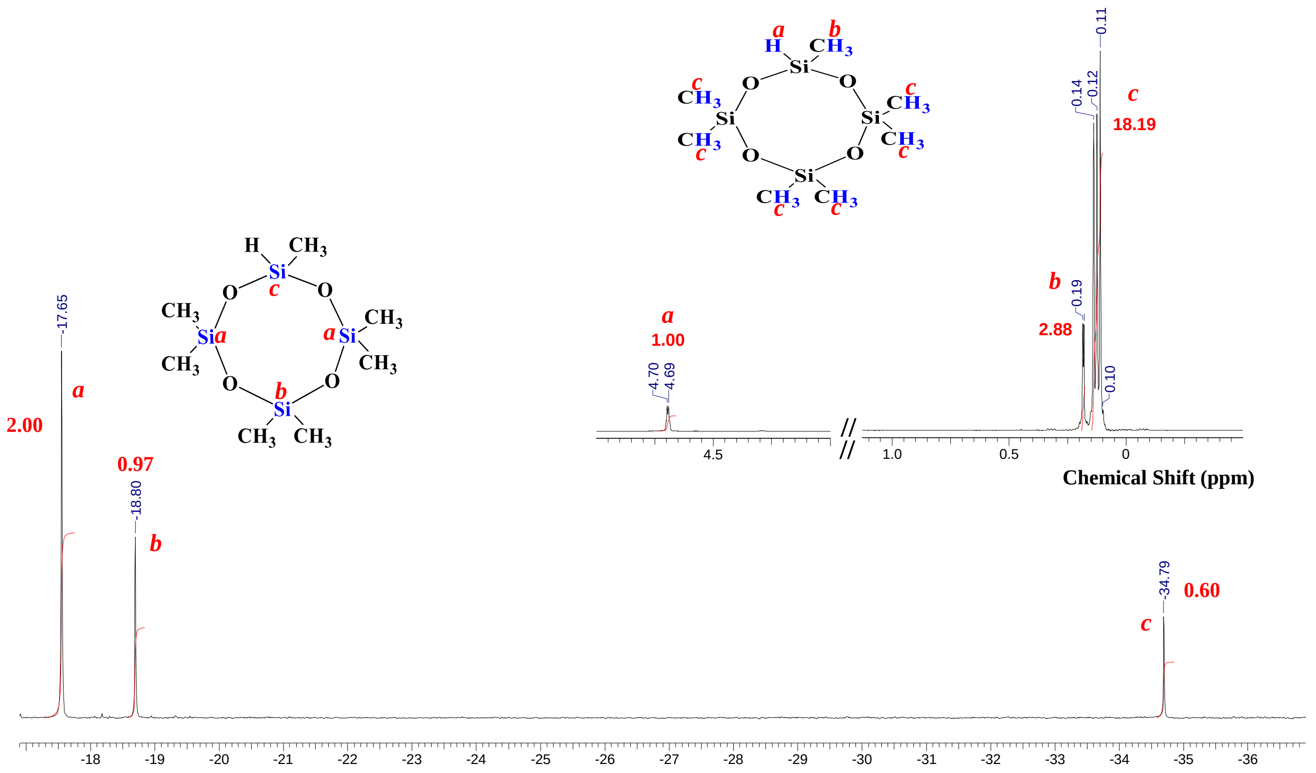

Figure S12. ^{1}H (top) and ^{29}Si (bottom) NMR spectra for 7-hydro-1,1,3,3,5,5,7-heptamethylcyclotetrasiloxane

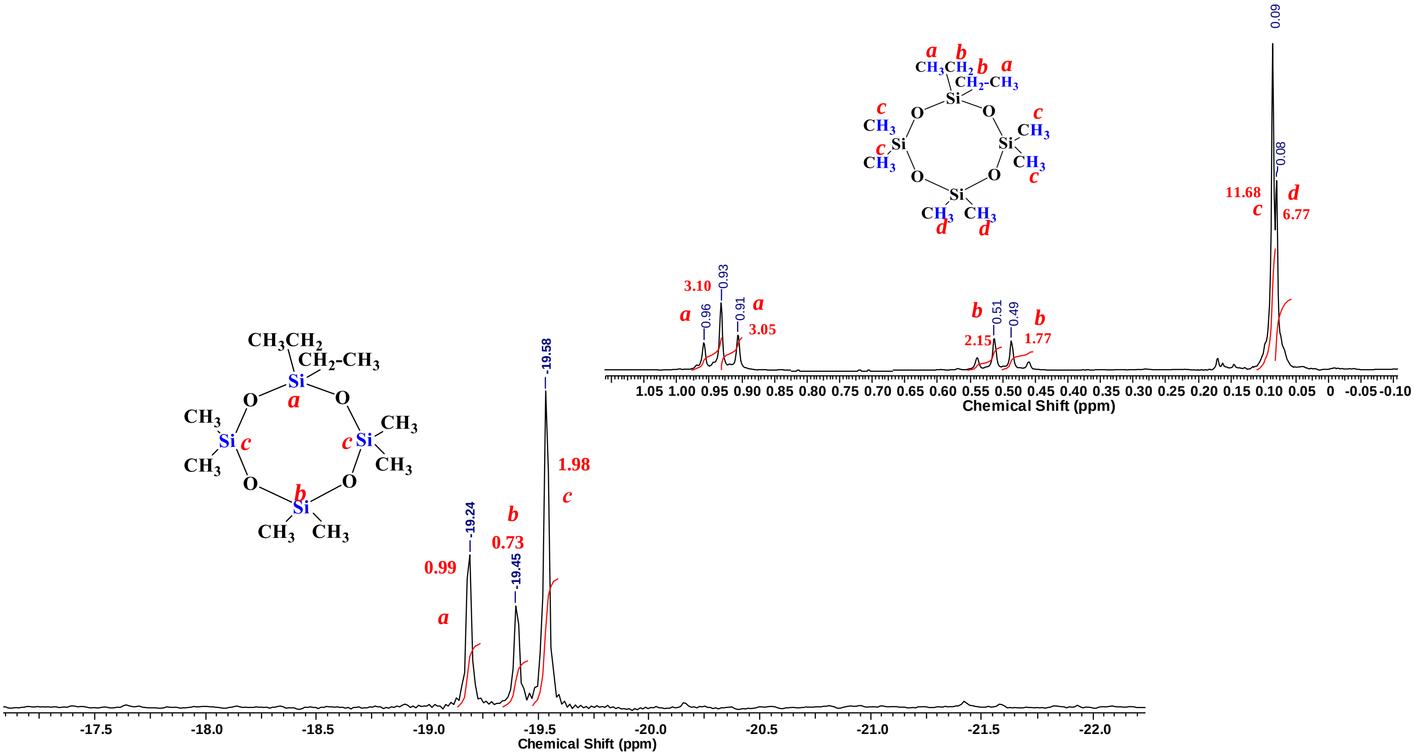

Figure S13. ^{1}H (top) and ^{29}Si (bottom) NMR spectra for 7,7-diethyl-1,1,3,3,5,5-hexamethylcyclotetrasiloxane

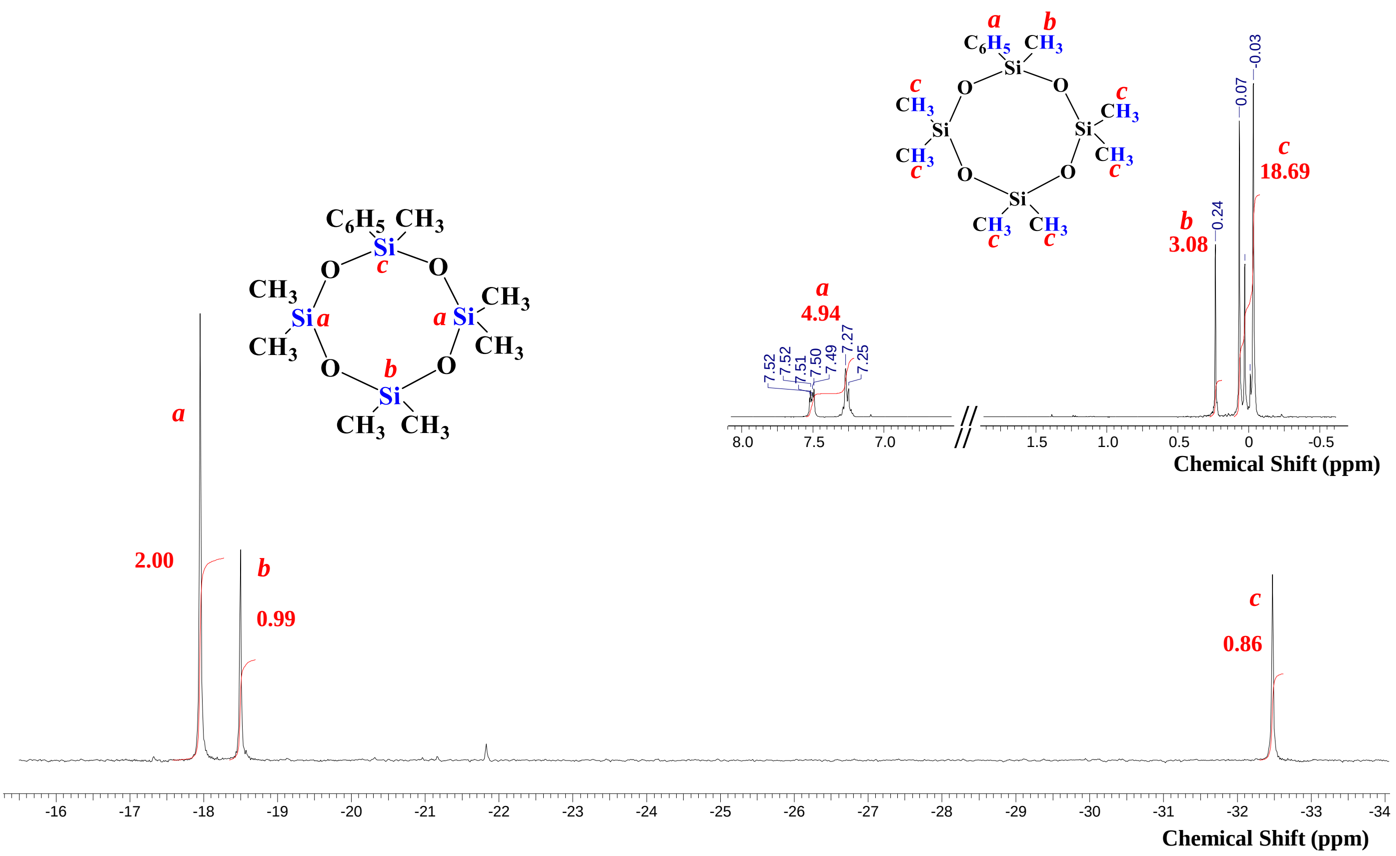

Figure S14. ^{1}H (top) and ^{29}Si (bottom) NMR spectra for 1,1,3,3,5,5,7-heptamethyl-7-phenylcyclotetrasiloxane

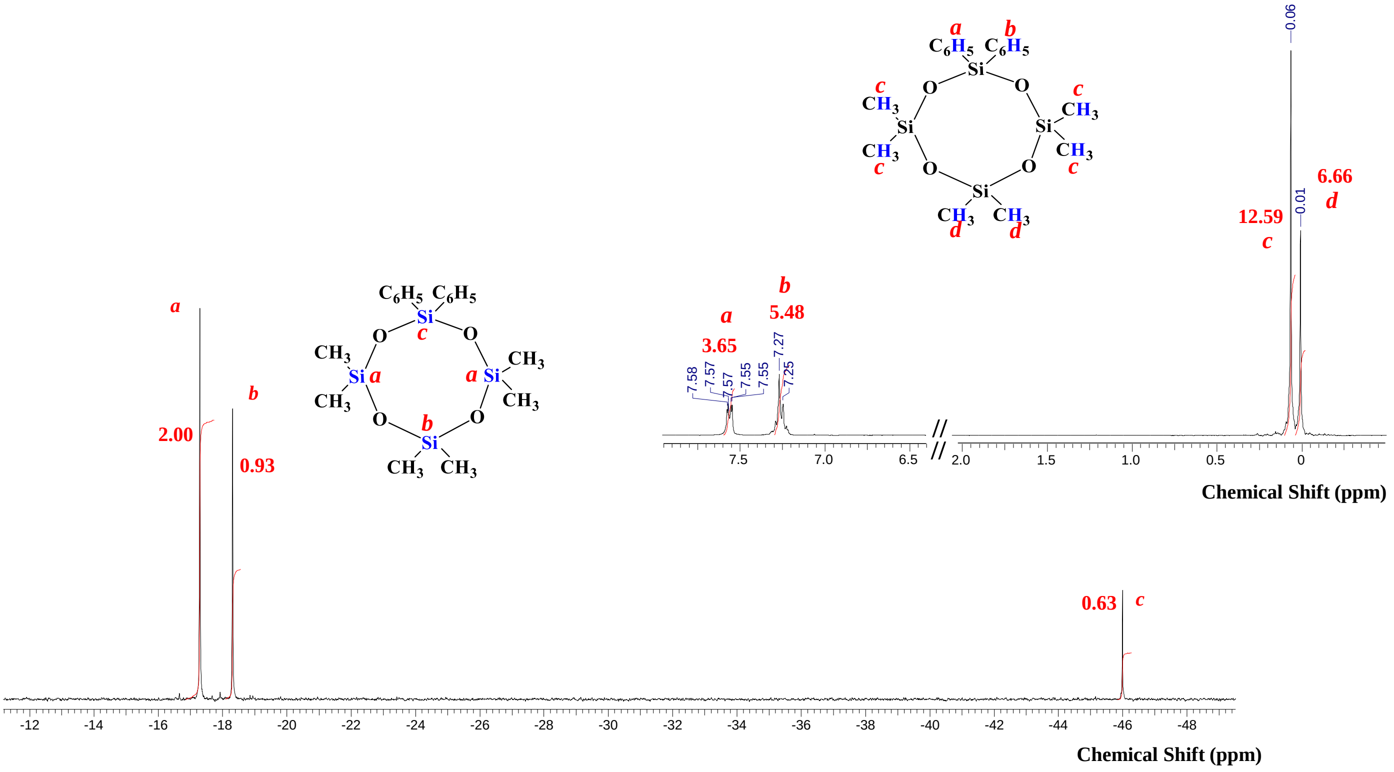

Figure S15. ^{1}H (top) and ^{29}Si (bottom) NMR spectra for 1,1,3,3,5,5-hexamethyl-7,7-diphenylcyclotetrasiloxane

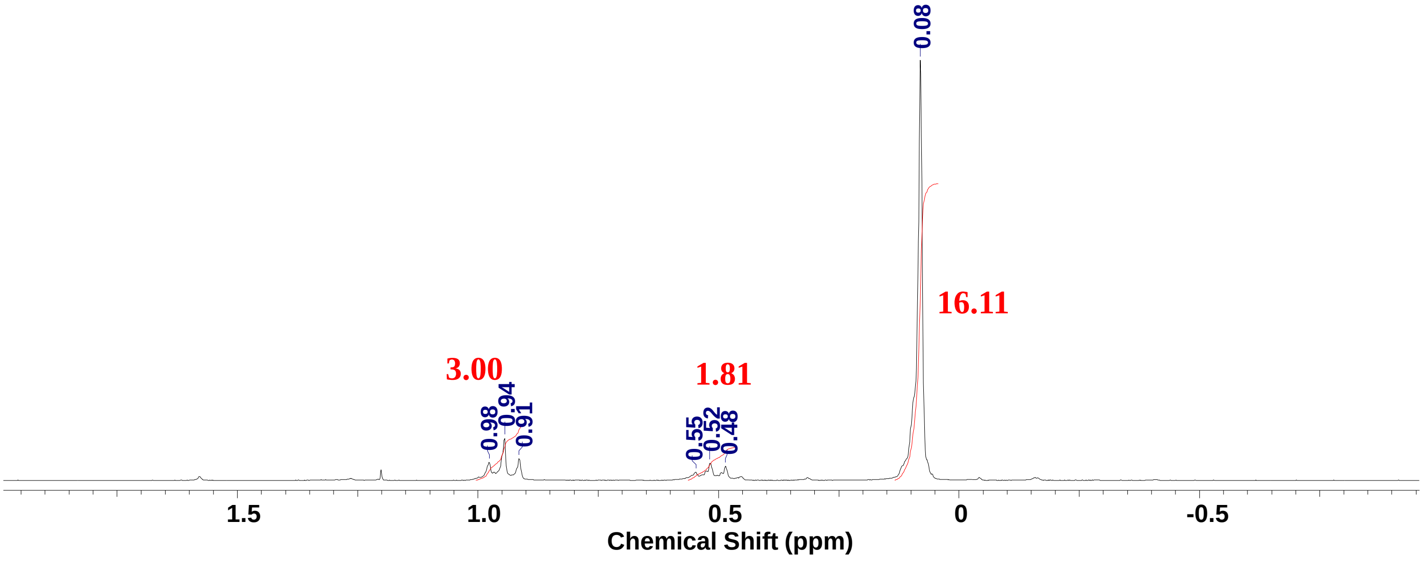

Figure S16. ^{1}H NMR spectra for copolymers from $[(D^{Me_2})_4]$ and $[(D^{Et_2})_4]$ (№ 1, Table 3)

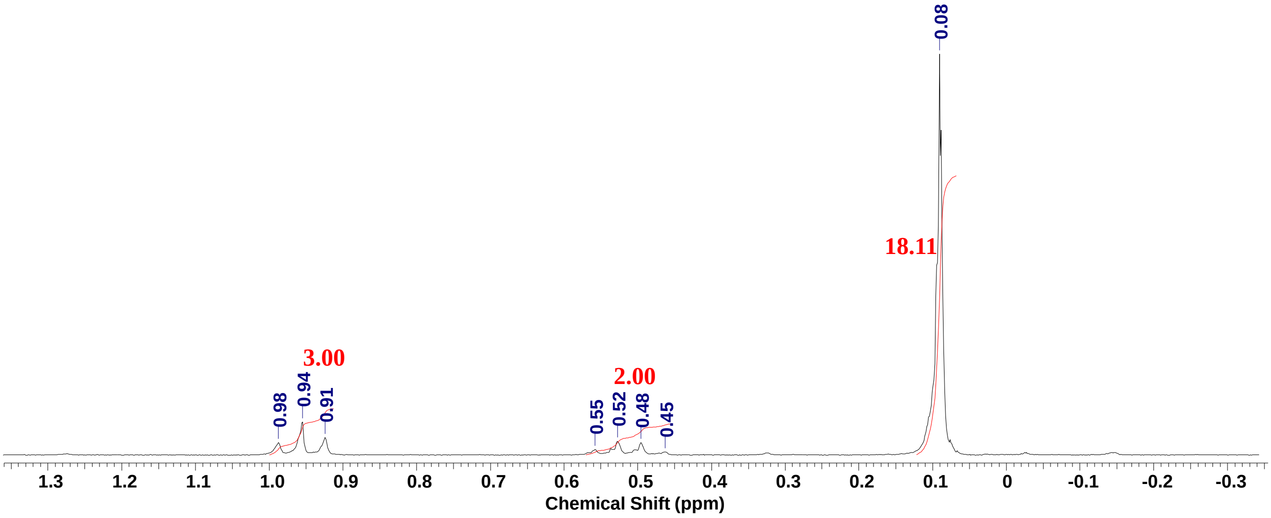

Figure S17. ^{1}H NMR spectra for copolymers from $[(D^{Me_2})_4]$ and $[(D^{Me_2})_3(D^{Et_2})]$ (№ 2, Table 3)

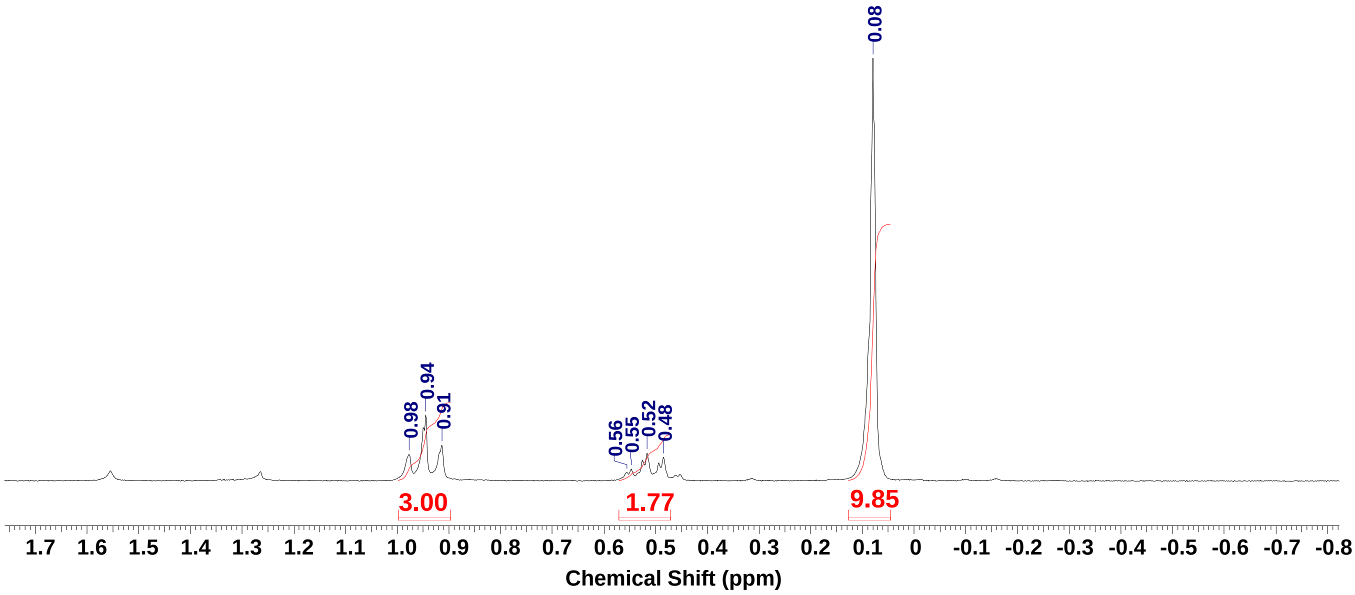

Figure S18. ^{1}H NMR spectra for copolymer from $[(D^{Me_2})_3(D^{Et_2})]$ (№ 3, Table 3)

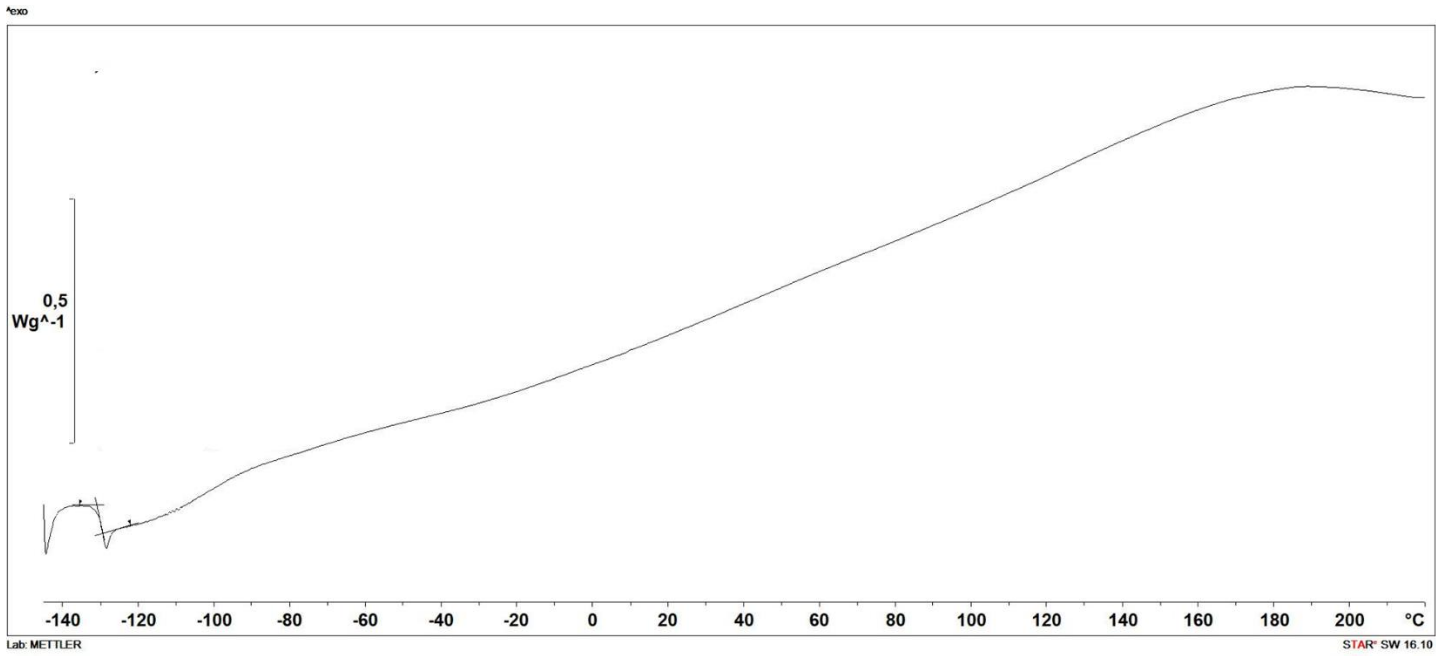

Figure S19. DSC curves of copolymer from $[(D^{Me_2})_4]$ and $[(D^{Me_2})_3(D^{Et_2})]$ (№2, Table 3)

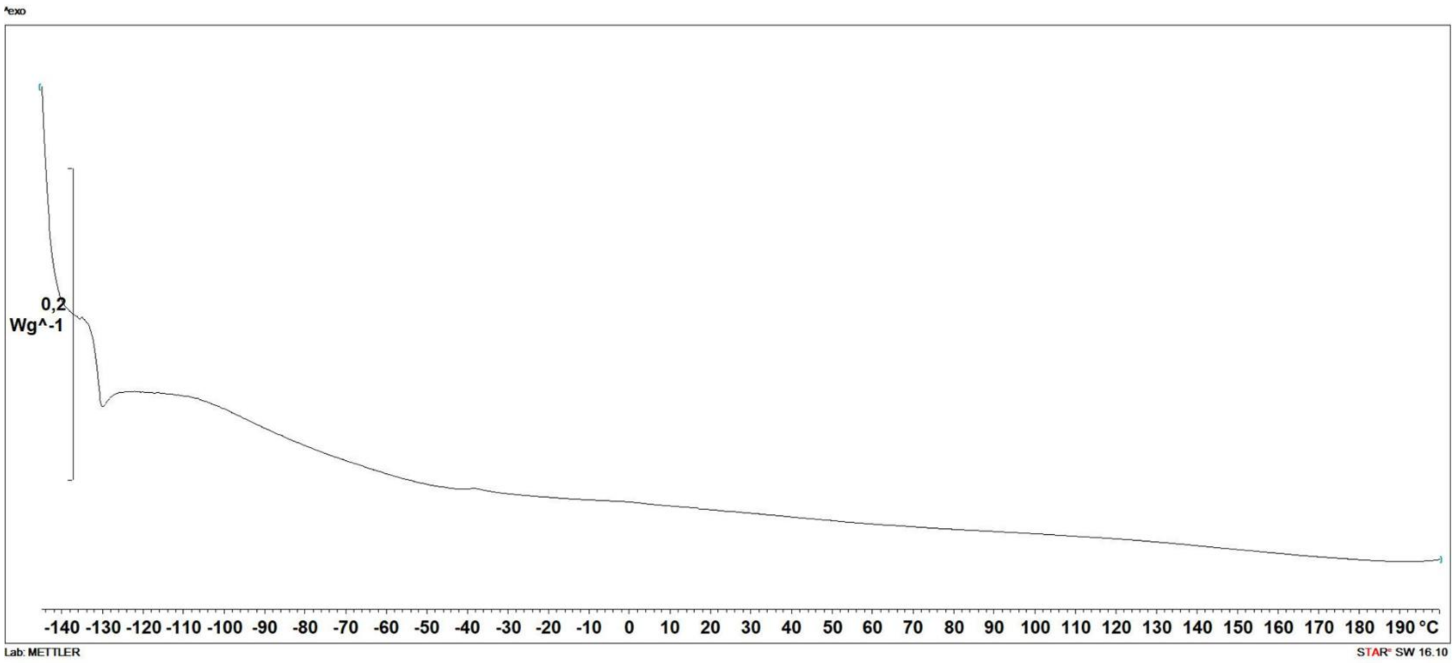

Figure S20. GPC curves of copolymer $[(D^{Me_2})_3(D^{Et_2})]$ (№3, Table 3)
